# Relative Age Effect in Elite German Soccer: Influence of Gender and Competition Level

**DOI:** 10.3389/fpsyg.2020.587023

**Published:** 2021-01-07

**Authors:** Martin Götze, Matthias W. Hoppe

**Affiliations:** ^1^Faculty of Sport Science, Institute of Movement and Training Science I, University of Leipzig, Leipzig, Germany; ^2^Institute for Applied Training Science, Leipzig, Germany

**Keywords:** birthdate, season of birth, female, football, sex differences, talent selection, expertise, team sport

## Abstract

The relative age effect (RAE) is associated with (dis)advantages in competitive sports. While the RAE in elite male soccer reveals a skewed birthdate distribution in relation to a certain cut-off date, research of RAE in elite female soccer is affected by small number of samples and conflicting results. The purpose of this study was to investigate the RAE in elite adult German soccer regarding gender and competition level. The sample comprised 680 female and 1,083 male players of the two top German leagues during the 2019/20 season and German national teams (A-Team to Under 19). Differences between the observed and expected birthdate distributions were analyzed using chi-square statistics and effect sizes followed by calculating odds ratios. Results showed a statistically significant RAE with small effect size across all players included for both genders (female players: *P* < 0.001, *W* = 0.16, male players: *P* < 0.001, *W* = 0.23). The identified RAE was based on an over-representation of players born at the beginning of the year. According to gender and competition level, RAEs were more pronounced in German male soccer. While significant RAEs were found among males in the first two leagues (first league: *P* < 0.001, *W* = 0.19, second league: *P* < 0.001, *W* = 0.26), the RAE of females was more pronounced in the second league (first league: *P* = 0.080, *W* = 0.16, second league: *P* = 0.002, *W* = 0.20). The analysis of RAE regarding the national teams revealed a statistically significant RAE with large effect size for only the youngest investigated age group of male players (Under 19: *P* = 0.022, *W* = 0.52). Our data show an RAE in female and male German adult soccer, which could be accompanied by a loss of valuable elite players during the youth phase of the career. Consequently, the pool of talented players at the adult level would be limited.

## Introduction

In sports, it has been shown that the relative age effect (RAE) favors relatively older athletes in terms of talent selection and development opportunities (Till et al., 2009). The term “relative age” refers to the difference in one’s birthdate in relation to a cut-off date used to group children and adolescents (Smith et al., 2018). For example, in an age-based system with January 1st as a cut-off date, an individual born in January is almost 1 year older than an individual born in December. Because the RAE can lead to selection bias due to physical, cognitive, psychological and social attributes, it may be considered as discrimination against athletes who are born later within an age cohort (Simmons and Paull, 2001; Dixon et al., 2011). In light of the fact that selection bias in children and youth sports could lead to non-selection and dropout of talents, the long-term consequences for professional sports are obvious (Baker et al., 2010).

Review studies and meta-analyses show that there are various factors moderating the RAE in sport (Cobley et al., 2009; Baker et al., 2010; Sierra-Díaz et al., 2017; Smith et al., 2018). These include age, sport context, competition level, gender, and playing position. One of the most notable moderators of RAE appears to be gender (Baker et al., 2010), although a comprehensive study of the RAE shows little evidence for gender influences (Cobley et al., 2009). This outcome is most likely related to the fact that only 2% of all included athletes are female. Therefore, a recent meta-analysis examined the prevalence and magnitude of the RAE specifically in female athletes (Smith et al., 2018). The findings show a statistically significant, but small RAE across all sport contexts with the relatively older of the age cohort were 25% more likely to be represented. In addition, the competition level moderates the RAE significantly, but with inconsistent effects in elite adult athletes. Thus, a sport-specific analysis of the RAE could provide more insights into the competition level of elite female athletes.

In elite adult soccer, the influence of gender on the RAE is also affected by the small number of female samples and conflicting results (Vincent and Glamser, 2006; Baker et al., 2009; Delorme et al., 2009b; Romann and Fuchslocher, 2011; Sedano et al., 2015). For example, there is no statistically significant RAE in elite female soccer in the US Soccer Federation (Vincent and Glamser, 2006), French professional championship (Delorme et al., 2009b), or the Swiss national teams (Romann and Fuchslocher, 2011). However, other studies have found a significant RAE (Baker et al., 2009; Sedano et al., 2015). In contrast, a detailed examination of the birthdate distribution per quarter reveals differences between the studies in Spanish and US soccer. In elite male soccer, on the other hand, there are numerous analyses of the RAE (Barnsley et al., 1992; Verhulst, 1992; Helsen et al., 1998; Musch and Hay, 1999; Cobley et al., 2008; Ostapczuk and Musch, 2013; Brustio et al., 2018; Yagüe et al., 2018; Kelly et al., 2020). Overall, these studies show a consistent RAE with an over-representation of players born at the beginning of their cohort year. Studies examining gender as a moderator of RAE in elite soccer with comparable female and male cohorts are rare and characterized by contradictory results. In U 18 European Championship, there is no significant difference in RAE between female and male players (Helsen et al., 2005). However, in US and Japanese soccer, there are gender differences of RAE due to a weaker skewed birthdate distribution in female players (Vincent and Glamser, 2006; Baker et al., 2009; Nakata and Sakamoto, 2012). More research is needed to investigate possible gender differences in elite adult soccer.

In Germany, soccer is a highly popular sport and the German Soccer Association has a high number of female (1,155,785) and male (6,016,151) members (German Soccer Association, 2019). The UEFA Club Coefficient shows that female and male German club teams are among the top three in the March Ranking 2020 (UEFA, 2020). Moreover, the national teams are ranked third in the aggregated women’s and men’s FIFA World Ranking (FIFA, 2019). To the best of our knowledge, no study has provided evidence of gender influences on the RAE in the highest competition levels from a leading soccer nation. Thus, this study aimed to investigate the RAE in female and male soccer players competing at the top two German leagues and German national teams (A-Team to Under (U) 19). Based on previous studies (Vincent and Glamser, 2006; Cobley et al., 2009; Schorer et al., 2009; Baker et al., 2010; Nakata and Sakamoto, 2012; Smith et al., 2018), it was hypothesized that there is an RAE in elite adult players, with the RAE being more prominent among males than females. Additionally, we assumed reduced RAE magnitudes on the highest competition level.

## Materials and Methods

### Subjects

Our study included a total sample of 680 female and 1,083 male adult soccer players, competing at the top two German leagues during the season 2019/20. Additionally, female and male rosters of the German national teams [A-Team, U 21 (male only), U 20, and U 19] were considered. The female U 21 national team could not be included, because this team was not managed by the German Soccer Association during the investigation period.

Because the rosters of the national teams varied depending on the importance of the meeting, only the rosters of the past two major tournaments (FIFA World Cup and UEFA European Championship) dating back to the year 2015 were analyzed. This process is in line with a previous study (Romann and Fuchslocher, 2013). Player birthdates were collected from the official data center of the German Soccer Association (accessed 7th April 2020)^1^. Players listed twice in national championships (eight females playing in the first and second league) were assigned to the team in which they participated more frequently. Because all data were freely available from the internet, no approval by an Ethical Committee was required. Table 1 gives an overview of the players included.

**TABLE 1:** Overview of the included female and male elite adult soccer players.

Gender	Subgroups	Competition level	Number of teams (N)	Number of players (N)	Mean chronological age and SD at the beginning of competition (years)
Female	1st League (Frauen-Bundesliga)	National Championship 2019/20	12	280	23.9 (4.2)
	2nd League (2. Frauen-Bundesliga)	National Championship 2019/20	14	400	21.0 (4.0)
	A-Team	FIFA Women’s World Cup 2019 and UEFA Women’s European Championship 2017	2	46	26.2 (3.3)
	U 20	FIFA U-20 Women’s World Cup 2018 and FIFA U-20 Women’s World Cup 2016	2	42	19.3 (0.9)
	U 19	UEFA U-19 Women’s European Championship 2019 and FIFA U-19 Women’s World Cup 2018	2	40	18.4 (0.6)
Male	1st League (Bundesliga)	National Championship 2019/20	18	540	25.2 (4.5)
	2nd League (2. Bundesliga)	National Championship 2019/20	18	543	25.2 (4.2)
	A-Team	FIFA World Cup 2018 and UEFA European Championship 2016	2	46	26.5 (3.4)
	U 21	UEFA U-21 European Championship 2019 and UEFA U-21 European Championship 2017	2	46	22.3 (0.9)
	U 20	FIFA U-20 World Cup 2017 and FIFA U-20 World Cup 2015	2	42	20.0 (0.3)
	U 19	UEFA U-19 European Championship 2017 and UEFA U-19 European Championship 2016	2	36	19.1 (0.3)

*U, under; SD, standard deviation.*

### Classification of Relative Age

The birth month of each player was used to define the birth quarter and half-year distribution per semester. To calculate the RAE, the annual age groups were broken down into quartiles (Cobley et al., 2009) and half-year groups (Cobley et al., 2008). The cut-off date was defined as January 1st. Therefore, the year was divided into four quartiles: Q1 = January, February and March, Q2 = April, May and June, Q3 = July, August and September, Q4 = October, November and December. Furthermore, the year was divided into two semesters: S1 = January to June, S2 = July to December. The observed birthdate distributions of all players were calculated four each quarter and semester. The expected birthdate distribution was calculated gender- and age-specifically (female age group: 1983–2003, male age group: 1978–2002) from the corresponding reference population of births in Germany and served as control (Delorme and Champely, 2015). These birthdate statistics were also publically available on the internet (Statistisches Bundesamt, 2020).

### Statistical Analyses

Chi-square goodness-of-fit tests were used to compare the observed and expected birthdate distributions. To determine subgroup differences, odds ratios (ORs) and 95% confidence intervals were calculated. The Chi-square goodness-of-fit tests were used to detect whether or not the assigned quartile of birth profile for a given sample was significantly different to an expected distribution (females: Q1 = 24.8%, Q2 = 24.8%, Q3 = 26.5%, Q4 = 23.9%, males: Q1 = 24.8%, Q2 = 25.0%, Q3 = 26.4%, Q4 = 23.9%). The ORs were calculated to test the odds of the frequency of a quarter/semester to another (Q1–Q3 vs. Q4, S1 vs. S2), whereby the Q4/S2 (relative youngest) functioned as the reference. An OR of 1.00 indicated that the frequency is equal in both quarters/semesters, while an OR of 2.00 indicated that the frequency of one quarter/semester is twice as high compared to the other (Smith et al., 2018).

Furthermore, effect sizes (Cohen’s W) were calculated to determine the magnitudes of the chi-square tests. According to Cohen (1988), *W* = 0.10, *W* = 0.30, and *W* = 0.50 indicated a small, medium, and large effect, respectively. An alpha level of *P <* 0.05 was set for statistical significance. All statistical analyses were computed with a statistical software package (SPSS, version 23.0, SPSS Inc. Chicago, IL, United States).

## Results

The birthdate distribution by quarter for each female and male subgroup is shown in Table 2. There was a statistically significant RAE for the subgroup of all female and male players with small effect sizes [all female players: *X*^2^ (3, *N* = 808) = 20.82, *P* < 0.001, *W* = 0.16, all male players: *X*^2^ (3, *N* = 1,253) = 68.42, *P* < 0.001, *W* = 0.23]. Players born at the beginning of the year were over-represented (all female players: Q1 vs. Q4, *OR* = 1.49, all male players: Q1 vs. Q4, *OR* = 2.00, Table 3) and the ORs declined for the comparisons later in the year with Q4 being inferior in each case (all female players: Q2 vs. Q4, *OR* = 1.18, Q3 vs. Q4, *OR* = 1.08, all male players: Q2 vs. Q4, *OR* = 1.56, Q3 vs. Q4, *OR* = 1.44).

**TABLE 2:** Birthdate distribution of the female and male elite adult soccer players compared to the corresponding reference population.

	Category	Q1	Q2	Q3	Q4	Total	*X*^2^	*P*	Cohen’s W	Effect
Female	RP (1983–2003)	2,037,237	2,040,780	2,181,311	1,968,470	8,227,798				
	(%)	24.8	24.8	26.5	23.9					
	1st League	83	72	57	68	280	6.75	0.080	0.16	Small
	(%)	29.6	25.7	20.4	24.3					
	2nd League	131	96	98	75	400	15.34	**0.002**	0.20	Small
	(%)	32.8	24.0	24.5	18.8					
	A-Team	13	11	9	13	46	1.44	0.697	0.18	Small
	(%)	28.3	23.9	19.6	28.3					
	U 20	14	14	7	7	42	4.92	0.178	0.34	Medium
	(%)	33.3	33.3	16.7	16.7					
	U 19	12	8	13	7	40	2.04	0.565	0.23	Small
	(%)	30.0	20.0	32.5	17.5					
	All players	253	201	184	170	808	20.82	**<0.001**	0.16	Small
	(%)	31.3	24.9	22.8	21.0					
Male	RP (1978–2002)	2,597,318	2,619,274	2,763,735	2,507,238	10,487,565				
	(%)	24.8	25.0	26.4	23.9					
	1st League	161	151	140	88	540	20.50	**<0.001**	0.19	Small
	(%)	29.8	28.0	25.9	16.3					
	2nd League	194	126	122	101	543	36.44	**<0.001**	0.26	Small
	(%)	35.7	23.2	22.5	18.6					
	A-Team	17	11	12	6	46	5.04	0.169	0.33	Medium
	(%)	37.0	23.9	26.1	13.0					
	U 21	18	12	10	6	46	6.48	0.090	0.38	Medium
	(%)	39.1	26.1	21.7	13.0					
	U 20	16	11	10	5	42	5.66	0.129	0.37	Medium
	(%)	38.1	26.2	23.8	11.9					
	U 19	13	14	6	3	36	9.59	**0.022**	0.52	Large
	(%)	36.1	38.9	16.7	8.3					
	All players	419	325	300	209	1,253	68.42	**<0.001**	0.23	Small
	(%)	33.4	25.9	23.9	16.7					

*Q, quarter; RP, reference population. Bold = statistically significant at P < 0.05.*

**TABLE 3:** Odds ratios of the birthdate distribution for the female and male elite adult soccer players.

	Category	OR comparisons, Q1–4 and S1–2 (95 % CI)			
		Q1 vs. Q4	Q2 vs. Q4	Q3 vs. Q4	S1 vs. S2
Female	RP (1983–2003)	1.04 (1.03–1.04)	1.04 (1.03–1.04)	1.11 (1.11–1.11)	0.98 (0.98–0.99)
	1st League	1.22 (0.77–1.94)	1.06 (0.66–1.70)	0.84 (0.52–1.36)	1.24 (0.89–1.73)
	2nd League	1.75 (1.18–2.60)	1.28 (0.85–1.93)	1.31 (0.87–1.97)	1.31 (0.99–1.73)
	A-Team	1.00 (0.33–3.03)	0.85 (0.27–2.63)	0.69 (0.22–2.23)	1.09 (0.48–2.47)
	U 20	2.00 (0.58–6.87)	2.00 (0.58–6.87)	1.00 (0.26–3.82)	2.00 (0.83–4.83)
	U 19	1.71 (0.48–6.17)	1.14 (0.30–4.37)	1.86 (0.52–6.61)	1.00 (0.42–2.40)
	All players	1.49 (1.13–1.96)	1.18 (0.89–1.57)	1.08 (0.81–1.44)	1.28 (1.05–1.56)
Male	RP (1978–2002)	1.04 (1.03–1.04)	1.05 (1.04–1.05)	1.10 (1.10–1.11)	0.99 (0.99–0.99)
	1st League	1.83 (1.29–2.60)	1.72 (1.20–2.45)	1.59 (1.11–2.28)	1.37 (1.08–1.74)
	2nd League	1.92 (1.37–2.69)	1.25 (0.88–1.78)	1.21 (0.85–1.72)	1.44 (1.13–1.82)
	A-Team	2.83 (0.83–9.67)	1.83 (0.51–6.60)	2.00 (0.56–7.09)	1.56 (0.68–3.56)
	U 21	3.00 (0.88–10.18)	2.00 (0.56–7.09)	1.67 (0.46–6.06)	1.88 (0.81–4.33)
	U 20	3.20 (0.86–11.81)	2.20 (0.57–8.47)	2.00 (0.51–7.80)	1.80 (0.75–4.32)
	U 19	4.33 (0.91–20.60)	4.67 (0.99–22.03)	2.00 (0.38–10.58)	3.00 (1.11–8.14)
	All players	2.00 (1.60–2.52)	1.56 (1.23–1.97)	1.44 (1.13–1.82)	1.46 (1.25–1.71)

*Q, quarter; S, semester; RP, reference population; OR, odds ratio; CI, confidence interval.*

Regarding the influence of gender, the half-year distribution of birthdates of all included players revealed higher OR of S1 vs. S2 for males than for females (*OR* = 1.46 vs. 1.28, Table 3). With the exception of the U 20 national team, males also had higher ORs of S1 vs. S2 compared to females for the respective competition level (first league: *OR* = 1.37 vs. 1.24, second league: *OR* = 1.44 vs. 1.31, A-Team: *OR* = 1.56 vs. 1.09, U 20: *OR* = 1.80 vs. 2.00, U 19: *OR* = 3.00 vs. 1.00). The OR of S1 vs. S2 for the reference population was 0.99 and 0.98 for males and females, respectively.

Analyzing the competition level of male players, the first two leagues of national championship showed significant RAEs with small effect sizes [first league: *X*^2^ (3, *N* = 540) = 20.50, *P* < 0.001, *W* = 0.19, second league: *X*^2^(3, *N* = 543) = 36.44, *P* < 0.001, *W* = 0.26]. In both leagues, players born during the first half of the year were over-represented (first league: *OR* = 1.37, second league: *OR* = 1.44), while the OR of Q1 vs. Q4 indicated a higher frequency of relatively older players in the second league (*OR* = 1.92) compared to the first league (*OR* = 1.83). In addition, the analyzed male U 19 national teams revealed a significant RAE with a large effect size [*X*^2^(3, *N* = 36) = 9.59, *P* = 0.022, *W* = 0.52]. Again, players born during the first half of the year were over-represented (S1 vs. S2, *OR* = 3.00). No significant RAEs were identified for the A-Team (*P* = 0.169), the U 21 (*P* = 0.090), or the U 20 team (*P* = 0.129).

Regarding the competition level of female players, there was only a significant RAE in the subgroup of the second league of the national championship with a small effect size [*X*^2^(3, *N* = 400) = 15.34, *P* = 0.002, *W* = 0.20]. A comparison of births over the half-year distribution showed an over-representation of the first 6 months for second league players (S1 vs. S2, *OR* = 1.31). Also, an OR of 1.75 of the comparison between Q1 vs. Q4 indicated that relatively older players of the second league were more likely to be represented. For all other female subgroups (first league and national teams), no statistically significant RAE was found (*P ≥* 0.080).

## Discussion

This study investigated the RAE in elite adult German soccer according to gender and competition level. Our main findings were: (a) There was a statistically significant RAE with small effect sizes across all included players for both genders, (b) the RAE was more prominent among males, and (c) while significant RAEs were found among males in the first two leagues, the RAE of females was more pronounced in the second league.

The overall RAE for all included soccer players showed that players born at the beginning of the year were consistently over-represented (Tables 2, 3). This result is in line with the RAE of male players reported in several elite soccer leagues worldwide (Helsen et al., 1998, 2012; Musch and Hay, 1999; Salinero et al., 2013; Brustio et al., 2018; Rađa et al., 2018; Doyle and Bottomley, 2019; Lupo et al., 2019). For female players, it is, however, only partially consistent with the literature (Vincent and Glamser, 2006; Delorme et al., 2009b; Romann and Fuchslocher, 2011; Sedano et al., 2015). In this context, it is evident that the RAE can be influenced by various attributes such as physical, cognitive, psychological, and social aspects (Cobley et al., 2009; Dixon et al., 2011; Brustio et al., 2018; Kelly and Williams, 2020). In particular, physical attributes are more prominent in contact sports, like soccer, than in noncontact sports (Delorme et al., 2009b). In fact, it has recently been shown that power-associated capacities differ between different age and starting-nonstarting youth players (Hoppe et al., 2020). Therefore, a skewed birth distribution of elite adult players could be based on a selection bias in youth soccer (Simmons and Paull, 2001). With this in mind, relatively older youth players could be more frequently selected for talent development programs due to their possible advantages in anthropometric development and power capacities (Brustio et al., 2018; Kelly and Williams, 2020). In previous research, it was assumed that in addition to an average higher level of maturity, relatively older youth players also receive more competition time. These players may benefit from greater support in performance development than their relatively younger counterparts (Helsen et al., 2012), which may support our assumptions.

Regarding the influence of gender, the overall RAE was more prominent among males (Table 2). The literature shows inconsistent results in a gender-specific comparison of the RAE in soccer. For example, Helsen et al. (2005) identified no RAEs for female and male soccer players of the U 18 European Championship. To the contrary, Vincent and Glamser (2006) showed gender differences of the RAE of 17-year-old elite soccer players with an RAE in males, but not in females. Possible explanations for gender-specific differences of the RAE can be related to the level of attraction of a sport for girls and boys, country-specific differences, and variations of competition levels (Baker et al., 2010). When the comparison of gender differences in the RAE is extended to other team sports, some research suggests a greater RAE magnitude in males than in females (Baker et al., 2009; Schorer et al., 2009; Nakata and Sakamoto, 2012). One reason for this, and possibly also for our results, could be a less intense competition within a team to make the starting line-up (Musch and Grondin, 2001; Lupo et al., 2019). For example, in a less popular sport, the number of available athletes is reduced and the demand for positions is not as great (Schorer et al., 2009). Therefore, it is expected to have a smaller RAE when fewer athletes participate in the sport. Other explanations may include a reduced depth of competition and the interaction of maturational and biological differences with socialization effects (Vincent and Glamser, 2006; Baker et al., 2010; Nakata and Sakamoto, 2012). However, to elucidate these speculations, more studies are needed.

Regarding the competition level, the RAE was more pronounced at the second leagues, even though there were statistically significant differences among males in the first league (Table 2). Also, in US, English, Italian, and French soccer, several studies concluded that a higher competition level leads to a lower RAE (Baker et al., 2009; Delorme et al., 2009a; Fleming and Fleming, 2012; Brustio et al., 2018). This effect could be explained by the increased importance of technical, tactical, and psychological aspects at the elite level (Rampinini et al., 2009; Carling, 2013; Vestberg et al., 2020). For example, a performance analysis of the first league in German male soccer showed that the physical match running performance alone is not a key indicator for achieving success (Hoppe et al., 2015). Conversely, a more athletic competition style in the second league could influence the RAE. A higher injury rate in the second compared to the first league of German male soccer is may be an indirect indicator for the higher physical demands (Klein et al., 2019). At the level of individuals, another possible explanation could be the so-called “underdog” hypothesis (Gibbs et al., 2012), where the relatively younger players may benefit from more competitive play with relatively older players at young ages. This may help the relatively younger players to reach greater resiliency and coping skills at an elite adult level. For instance, there was a strong RAE within an English professional soccer academy, whereby relatively older players were three times more likely to be recruited compared to relatively younger players (Kelly et al., 2020). However, when analyzing the conversion rate, relatively younger players were four times more likely to achieve a professional contract once they have been selected for the academy. This is potentially achieved by overcoming major challenges in training and competition resulting in enhanced resilience (Kelly and Williams, 2020). Finally, in relation to the RAE of male players of the second league in our study, comparable results were obtained across the top five European second leagues in male soccer (Rađa et al., 2018). The authors attributed this observation to the fact that there is no second chance to enter the first league for late-born players.

Our findings in female players, namely that there was an RAE in the second but not in the first league, are partly in line with the literature. Similarly, in Spanish female soccer, a statistically significant RAE was found in second league players (Sedano et al., 2015). In contrast to our study, significant RAEs were also identified in the first Spanish league. In the US Soccer Federation (Vincent and Glamser, 2006) and the French professional championship (Delorme et al., 2009b), no significant RAEs were observed for females. One potential explanation for these discrepancies is that there are country-specific differences in the RAE of female soccer players, potentially due to different youth development and competition systems (Sierra-Díaz et al., 2017). For example, in German youth soccer, girls are allowed to compete with boys up to 16 years (German Soccer Association, 2020). This means that athletically stronger and presumably relatively older girls may be able to withstand this mixed-gender competition. In addition, in most German regional associations, it is allowed that girls of an older chronological age group can compete with boys of a younger chronological age group, which could intensify the RAE (Reinders, 2017).

A hypothetical explanation for the RAE of female second league players of our study could be due to structural reform. Starting with the 2018/19 season, the German second female soccer league was no longer divided into North and South. Instead, it was played in a single division with a reduced number of teams (German Soccer Association, 2017). This may has intensified the competition for making the team. Another potential reason could be the transition from youth to senior level (Lupo et al., 2019). Since the female youth players move directly to the senior leagues after the U 16/U 17, we assumed that the likelihood of relatively older players making this transition is higher, at least in part, to the second league. The difference in the average age between the first and second league (first league 23.9 ± 4.2 years, second league: 21.0 ± 4.0 years, Table 1) suggests that more female youth players make the transition to the second league initially. Possible advantages of the physical maturation of relatively older players may be a cause of the RAE in the second league, at least researchers trace RAE in male athletes to this effect (Vincent and Glamser, 2006; Lupo et al., 2019). To clarify also these assumptions, more research is needed.

Our analysis of the RAE regarding the national teams only showed an over-representation of players born at the beginning of the year for the youngest investigated age group of male players (U 19). There is evidence that the RAE decreases with age after adolescence (Cobley et al., 2009; Brustio et al., 2018). For the German national male youth soccer teams, Skorski et al. (2016) also identified a decreasing RAE with increasing age categories from U 16 to the senior A-Team. The authors suspected that this phenomenon could be related to a higher proportion of relatively younger players achieving a professional level (Skorski et al., 2016). It is worth mentioning that for national teams the best players were selected. After adolescence, when the advantages of early physical maturity fade away and the best players all have a similar physiological level, technical-tactical skills, and cognitive abilities could make the difference to produce successful outcomes. This might enable relatively younger players be more competitive than their relatively older counterparts (McCarthy and Collins, 2014).

## Limitations

Firstly, we did not record anthropometric and performance variables of the players, which may better describe the phenomenon of RAE. The discussion about physical advantages of players born close to the beginning of the age cohort is therefore somewhat speculative and thus more research is required. Secondly, the birthdates of players of national championships refer to only one season. This makes it difficult to draw conclusion about the development of an RAE over several seasons. Thirdly, we included national and international players of national championships, which could have an impact on the RAE magnitudes between the analyzed leagues (Schorer et al., 2009). Finally, the sample size in national teams is low. However, to solve that point, we grouped all players in a specific subgroup according to gender. In terms of statistics, we used the birth month of each player to define the birth quarter and half-year distribution per semester. It should be noted that this approach has, however, some limitations. For example, a player born on the 1st of January and a player born on the 31th of March are considered as equivalent. Decimal ranging or Poisson regression modeling could improve the sensitivity in quantifying the RAEs (Brustio et al., 2018; Gerdin et al., 2018; Doyle and Bottomley, 2019). Future studies should examine the influence of gender and competition level on the RAE in elite soccer over several seasons and possible causal factors of RAE such as physical maturation, anthropometric variables and individual performance for female and male players.

## Practical Implications

Previous studies have made recommendations such as yearly rotation of the cut-off date (Romann and Fuchslocher, 2011), shifting the start of talent selection to the period after puberty (Güllich, 2014; Güllich and Emrich, 2014), and education of the long-term vision of coaches in the talent selection process (Yagüe et al., 2018), which may all aim to mitigate the negative consequences of the RAE. A pragmatic approach during scouting at junior competitions can be age-ordered shirt numbering, which reduces the selection bias associated with the RAE, and thus also long-term consequences of the overall quality of the top senior teams (Mann and van Ginneken, 2017). The transition from youth soccer to senior leagues is of particular importance for the career of a talented player. Considerable age differences can occur, especially in female soccer, which may induce a RAE. In flexible match schedules, players from the oldest junior and youngest senior age groups could be combined for competitions aiming to bridge the gap during the transition and could reduce the RAE.

## Conclusion

In conclusion, our results demonstrate that RAEs exist in German elite adult female and male soccer. The statistically significant RAE of this study is based on an over-representation of players born at the beginning of the year. Considering the effect sizes and ORs, RAEs were more pronounced in males, although, similar effects were found among females in the second league. An RAE in adult soccer could be accompanied by a loss of valuable players during the youth phase of the career and limiting the pool of talented players at the adult level. In addition to explanations for the RAE in previous studies (Musch and Hay, 1999; Baker et al., 2009; Cobley et al., 2009; Helsen et al., 2012; Brustio et al., 2018; Smith et al., 2018), it is conceivable that the demanding transition from U 16/U 17 to the senior level of German female soccer intensify the RAE in this cohort. Coaches and practitioners should try to find solutions to assist players in the transition from youth to senior professional level. Depending on the organizational structures of the youth development system, flexible match schedules could possibly reduce the RAE of female players in this study.

## Data Availability Statement

Publicly available datasets were analyzed in this study. This data can be found here: https://datencenter.dfb.de/datencenter provided by German Soccer Association.

## Author Contributions

MG and MH: conceptualization and investigation. MG: data curation, formal analysis, methodology, project administration, visualization, and writing—original draft. MH: supervision and writing—review and editing. Both authors contributed to the article and approved the submitted version.

## Conflict of Interest

The authors declare that the research was conducted in the absence of any commercial or financial relationships that could be construed as a potential conflict of interest.

## Footnotes

1 https://datencenter.dfb.de/datencenter

## Abbreviations

RAE: relative age effect
OR: odds ratio.

## References

[B1] Baker J. Schorer J. Cobley S. (2010). Relative age effects: an inevitable consequence of elite sport? *Sportwissenschaft* 40 26–30. 10.1007/s12662-009-0095-2

[B2] Baker J. Schorer J. Cobley S. Bräutigam H. Büsch D. (2009). Gender, depth of competition and relative age effects in team sports. *Asian J. Exerc. Sports Sci.* 6 1–7. 10.1111/j.1600-0838.2008.00838.x 18627551

[B3] Barnsley R. H. Thompson A. H. Legault P. (1992). Family planning: football style. The relative age effect in football. *Int. Rev. Sociol. Sport* 27 77–87. 10.1177/101269029202700105

[B4] Brustio P. R. Lupo C. Ungureanu A. N. Frati R. Rainoldi A. Boccia G. (2018). The relative age effect is larger in Italian soccer top-level youth categories and smaller in Serie A. *PLoS One* 13:e0196253. 10.1371/journal.pone.0196253 29672644 PMC5909613

[B5] Carling C. (2013). Interpreting physical performance in professional soccer match-play: should we be more pragmatic in our approach? *Sports Med.* 43 655–663. 10.1007/s40279-013-0055-8 23661303

[B6] Cobley S. Baker J. Wattie N. McKenna J. (2009). Annual age-grouping and athlete development: a meta-analytical review of relative age effects in sport. *Sports Med.* 39 235–256. 10.2165/00007256-200939030-00005 19290678

[B7] Cobley S. Schorer J. Baker J. (2008). Relative age effects in professional German soccer: a historical analysis. *J. Sports Sci.* 26 1531–1538. 10.1080/02640410802298250 19040189

[B8] Cohen J. (1988). *Statistical Power Analysis for the Behavioral Sciences.* Hillsdale: Lawrence Erlbaum Associates.

[B9] Delorme N. Boiché J. Raspaud M. (2009a). Relative age effect in female sport: a diachronic examination of soccer players: RAE in French female soccer. *Scand. J. Med. Sci. Sports* 20 509–515. 10.1111/j.1600-0838.2009.00979.x 19602186

[B10] Delorme N. Boiché J. Raspaud M. (2009b). The relative age effect in elite sport: the french case. *Res. Q. Exerc. Sport* 80 336–344. 10.1080/02701367.2009.10599568 19650399

[B11] Delorme N. Champely S. (2015). Relative age effect and chi-squared statistics. *Int. Rev. Sociol. Sport* 50 740–746. 10.1177/1012690213493104

[B12] Dixon J. Horton S. Weir P. (2011). Relative age effects: implications for leadership development. *Int. J. Sport Soc.* 2 1–15. 10.18848/2152-7857/cgp/v02i02/54068

[B13] Doyle J. R. Bottomley P. A. (2019). The relative age effect in European elite soccer: a practical guide to poisson regression modelling. *PLoS One* 14:e0213988. 10.1371/journal.pone.0213988 30943241 PMC6447143

[B14] FIFA (2019). *FIFA Women’s Survey Report - Confederations & Global MAs.* Zurich: FIFA.

[B15] Fleming J. Fleming S. (2012). Relative age effect amongst footballers in the English Premier League and English Football League, 2010-2011. *Int. J. Perform. Anal. Sport* 12 361–372. 10.1080/24748668.2012.11868604

[B16] Gerdin G. Hedberg M. Hageskog C.-A. (2018). Relative age effect in swedish male and female tennis players born in 1998–2001. *Sports* 6:38. 10.3390/sports6020038 29910342 PMC6026833

[B17] German Soccer Association (2017). *2. Frauen-Bundesliga ab 2018 Eingleisig.* Frankfurt: German Soccer Association.

[B18] German Soccer Association (2019). *Mitgliederstatistik 2019.* Frankfurt: German Soccer Association.

[B19] German Soccer Association (2020). *Jugendordnung.* Frankfurt: German Soccer Association.

[B20] Gibbs B. G. Jarvis J. A. Dufur M. J. (2012). The rise of the underdog? The relative age effect reversal among Canadian-born NHL hockey players: a reply to Nolan and Howell. *Int. Rev. Sociol. Sport* 47 644–649. 10.1177/1012690211414343

[B21] Güllich A. (2014). Selection, de-selection and progression in German football talent promotion. *Eur. J. Sport Sci.* 14 530–537. 10.1080/17461391.2013.858371 24245783

[B22] Güllich A. Emrich E. (2014). Considering long-term sustainability in the development of world class success. *Eur. J. Sport Sci.* 14 S383–S397. 10.1080/17461391.2012.706320 24444233

[B23] Helsen W. F. Baker J. Michiels S. Schorer J. Van winckel J. Williams A. M. (2012). The relative age effect in European professional soccer: did ten years of research make any difference? *J. Sports Sci.* 30 1665–1671. 10.1080/02640414.2012.721929 23005576

[B24] Helsen W. F. Starkes J. L. Van Winckel J. (1998). The influence of relative age on success and dropout in male soccer players. *Am. J. Hum. Biol. Off. J. Hum. Biol. Assoc.* 10 791–798. 10.1002/(sici)1520-6300(1998)10:6<791::aid-ajhb10>3.0.co;2-1 28561412

[B25] Helsen W. F. van Winckel J. Williams A. M. (2005). The relative age effect in youth soccer across Europe. *J. Sports Sci.* 23 629–636. 10.1080/02640410400021310 16195011

[B26] Hoppe M. Slomka M. Baumgart C. Weber H. Freiwald J. (2015). Match running performance and success across a season in german bundesliga soccer teams. *Int. J. Sports Med.* 36 563–566. 10.1055/s-0034-1398578 25760152

[B27] Hoppe M. W. Barnics V. Freiwald J. Baumgart C. (2020). Contrary to endurance, power associated capacities differ between different aged and starting-nonstarting elite junior soccer players. *PLoS One* 15:e0232118. 10.1371/journal.pone.0232118 32343716 PMC7188207

[B28] Kelly A. L. Williams C. A. (2020). Physical characteristics and the talent identification and development processes in male youth soccer: a narrative review. *Strength Cond. J.* 42 15–34. 10.1519/SSC.0000000000000576

[B29] Kelly A. L. Wilson M. R. Gough L. A. Knapman H. Morgan P. Cole M. (2020). A longitudinal investigation into the relative age effect in an English professional football club: exploring the ‘underdog hypothesis.’. *Sci. Med. Footb.* 4 111–118. 10.1080/24733938.2019.1694169

[B30] Klein C. Bloch H. Burkhardt K. Kühn N. Schäfer M. (2019). *VBG-Sportreport 2019 – Analyse des Unfallgeschehens in den Zwei Höchsten Ligen der Männer: Basketball, Eishockey, Fußball, Handball. Eine Längsschnittbetrachtung drei Aufeinanderfolgender Spielzeiten.* Hamburg: VBG.

[B31] Lupo C. Boccia G. Ungureanu A. N. Frati R. Marocco R. Brustio P. R. (2019). The beginning of senior career in team sport is affected by relative age effect. *Front. Psychol.* 10:1465. 10.3389/fpsyg.2019.01465 31293489 PMC6606777

[B32] Mann D. L. van Ginneken P. J. M. A. (2017). Age-ordered shirt numbering reduces the selection bias associated with the relative age effect. *J. Sports Sci.* 35 784–790. 10.1080/02640414.2016.1189588 27238077

[B33] McCarthy N. Collins D. (2014). Initial identification & selection bias versus the eventual confirmation of talent: evidence for the benefits of a rocky road? *J. Sports Sci.* 32 1604–1610. 10.1080/02640414.2014.908322 24857164

[B34] Musch J. Grondin S. (2001). Unequal competition as an impediment to personal development: a review of the relative age effect in sport. *Dev. Rev.* 21 147–167. 10.1006/drev.2000.0516

[B35] Musch J. Hay R. (1999). The relative age effect in soccer: cross-cultural evidence for a systematic discrimination against children born late in the competition year. *Sociol. Sport J.* 16 54–64. 10.1123/ssj.16.1.54

[B36] Nakata H. Sakamoto K. (2012). Sex differences in relative age effects among Japanese athletes. *Percept. Mot. Skills* 115 179–186. 10.2466/10.05.17.PMS.115.4.179-186 23033755

[B37] Ostapczuk M. Musch J. (2013). The influence of relative age on the composition of professional soccer squads. *Eur. J. Sport Sci.* 13 249–255. 10.1080/17461391.2011.606841 23679141

[B38] Rađa A. Padulo J. Jelaska I. Ardigò L. P. Fumarco L. (2018). Relative age effect and second-tiers: no second chance for later-born players. *PLoS One* 13:e0201795. 10.1371/journal.pone.0201795 30089178 PMC6082547

[B39] Rampinini E. Impellizzeri F. M. Castagna C. Coutts A. J. Wisløff U. (2009). Technical performance during soccer matches of the Italian Serie A league: effect of fatigue and competitive level. *J. Sci. Med. Sport* 12 227–233. 10.1016/j.jsams.2007.10.002 18083631

[B40] Reinders H. (2017). *Strukturelle Förderung des Mädchenfußballs: Rechtliche Regelungen zum Jahrgangsversetzten Einsatz von Juniorinnen im Junioren-Fußball in den Landesverbänden des DFB.* Würzburg: Schriftenreihe des Nachwuchsförderzentrums für Juniorinnen.

[B41] Romann M. Fuchslocher J. (2011). Influence of the selection level, age and playing position on relative age effects in Swiss women’s soccer. *Talent Dev. Excell.* 3 239–247.

[B42] Romann M. Fuchslocher J. (2013). Influences of player nationality, playing position, and height on relative age effects at women’s under-17 FIFA World Cup. *J. Sports Sci.* 31 32–40. 10.1080/02640414.2012.718442 22909307

[B43] Salinero J. J. Pérez B. Burillo P. Lesma M. L. (2013). Relative age effect in European professional football. Analysis by position. *J. Hum. Sport Exerc.* 8 966–973. 10.4100/jhse.2013.84.07

[B44] Schorer J. Cobley S. Büsch D. Bräutigam H. Baker J. (2009). Influences of competition level, gender, player nationality, career stage and playing position on relative age effects. *Scand. J. Med. Sci. Sports* 19 720–730. 10.1111/j.1600-0838.2008.00838.x 18627551

[B45] Sedano S. Vaeyens R. Redondo J. C. (2015). The relative age effect in Spanish female soccer players. Influence of the competitive level and a playing position. *J. Hum. Kinet.* 46 129–137. 10.1515/hukin-2015-0041 26240656 PMC4519203

[B46] Sierra-Díaz M. González-Víllora S. Pastor-Vicedo J. Serra-Olivares J. (2017). Soccer and relative age effect: a walk among elite players and young players. *Sports* 5:5. 10.3390/sports5010005 29910365 PMC5969014

[B47] Simmons C. Paull G. C. (2001). Season-of-birth bias in association football. *J. Sports Sci.* 19 677–686. 10.1080/02640410152475801 11522143

[B48] Skorski S. Skorski S. Faude O. Hammes D. Meyer T. (2016). The relative age effect in elite German youth soccer: implications for a successful career. *Int. J. Sports Physiol. Perform.* 11 370–376. 10.1123/ijspp.2015-0071 26308489

[B49] Smith K. L. Weir P. L. Till K. Romann M. Cobley S. (2018). Relative age effects across and within female sport contexts: a systematic review and meta-analysis. *Sports Med.* 48 1451–1478. 10.1007/s40279-018-0890-8 29536262

[B50] Statistisches Bundesamt (2020). *Lebendgeborene: Deutschland, Jahre, Geschlecht.* Atlanta, GA: Statistisches Bundesamt.

[B51] Till K. Cobley S. Wattie N. O’Hara J. Cooke C. Chapman C. (2009). The prevalence, influential factors and mechanisms of relative age effects in UK rugby league: relative age effects in rugby league. *Scand. J. Med. Sci. Sports* 20 320–329. 10.1111/j.1600-0838.2009.00884.x 19486487

[B52] UEFA (2020). *UEFA Club Coefficients.* Nyon: UEFA.

[B53] Verhulst J. (1992). Seasonal birth distribution of west European soccer players: a possible explanation. *Med. Hypothes.* 38 346–348. 10.1016/0306-9877(92)90030-G 1491636

[B54] Vestberg T. Jafari R. Almeida R. Maurex L. Ingvar M. Petrovic P. (2020). Level of play and coach-rated game intelligence are related to performance on design fluency in elite soccer players. *Sci. Rep.* 10:9852. 10.1038/s41598-020-66180-w 32587269 PMC7316809

[B55] Vincent J. Glamser F. D. (2006). Gender differences in the relative age effect among US olympic development program youth soccer players. *J. Sports Sci.* 24 405–413. 10.1080/02640410500244655 16492604

[B56] Yagüe J. M. de la Rubia A. Sánchez-Molina J. Maroto-Izquierdo S. Molinero O. (2018). The relative age effect in the 10 best leagues of male professional football of the Union of European Football Associations (UEFA). *J. Sports Sci. Med.* 17 409–416. 30116114 PMC6090398

